# Navigating the unknown: assessing anthropogenic threats to beaked whales, family Ziphiidae

**DOI:** 10.1098/rsos.240058

**Published:** 2024-04-17

**Authors:** Laura J. Feyrer, Joy E. Stanistreet, Hilary B. Moors-Murphy

**Affiliations:** ^1^ Bedford Institute of Oceanography, Fisheries and Oceans Canada, Dartmouth, Nova Scotia B2Y 4A2, Canada; ^2^ Department of Biology, Dalhousie University, Halifax, Nova Scotia B3H 4R2, Canada

**Keywords:** pathway of effects, anthropogenic threats, beaked whales, cumulative impacts, marine conservation, conservation priorities

## Abstract

This review comprehensively evaluates the impacts of anthropogenic threats on beaked whales (Ziphiidae)—a taxonomic group characterized by cryptic biology, deep dives and remote offshore habitat, which have challenged direct scientific observation. By synthesizing information published in peer-reviewed studies and grey literature, we identified available evidence of impacts across 14 threats for each Ziphiidae species. Threats were assessed based on their pathways of effects on individuals, revealing many gaps in scientific understanding of the risks faced by beaked whales. By applying a comprehensive taxon-level analysis, we found evidence that all beaked whale species are affected by multiple stressors, with climate change, entanglement and plastic pollution being the most common threats documented across beaked whale species. Threats assessed as having a serious impact on individuals included whaling, military sonar, entanglement, depredation, vessel strikes, plastics and oil spills. This review emphasizes the urgent need for targeted research to address a range of uncertainties, including cumulative and population-level impacts. Understanding the evidence and pathways of the effects of stressors on individuals can support future assessments, guide practical mitigation strategies and advance current understanding of anthropogenic impacts on rare and elusive marine species.

## 1. Introduction

There are currently 24 recognized species of beaked whales in the family Ziphiidae, which primarily occur in remote offshore areas and waters exceeding 500 m depth [1]. This diversity reflects an evolving scientific understanding of the group, with new species classifications continuing to emerge over the last decade [2–4]. With the exception of Cuvier’s beaked whales (*Ziphius cavirostris*), northern bottlenose whales (*Hyperoodon ampullatus*), Baird’s beaked whales (*Berardius bairdii*) and Blainville’s beaked whales (*Mesoplodon densirostris*), there are limited data on the population structure or abundance for most species of beaked whales [5–9]. Eight species of beaked whales are currently assessed as Data Deficient by the International Union for the Conservation of Nature (IUCN [10]), highlighting how the notoriously data-poor status of this family of whales continues to challenge scientific understanding as well as their conservation. However, over the last two decades, prompted by mass strandings and a recognition of the negative effects of military sonar, expanding research efforts and long-term studies have increased our knowledge of beaked whale populations around the world [8]. While five species or subspecies have been assessed by the IUCN as Vulnerable, Near Threatened or Endangered, the extent of human threats affecting the diverse array of beaked whale species remains largely uncertain [10].

The primary objectives of this review were to compile available information on anthropogenic threats for all species of beaked whales and describe the pathways through which these stressors can cause adverse effects. Secondary objectives were to identify which species have been exposed or affected, and assess each threat for its potential level of impact given evidence across beaked whale species. We evaluate available scientific research and highlight gaps and uncertainties, with the aim of supporting evidence-based management, mitigation and prioritization of future studies that will improve the science-based conservation of all beaked whales.

## 2. Methods

Threats were initially identified from beaked whale status assessments [10–12] and classified into six categories based on threat source and attribute, similar to Avila *et al*. [13]. We reviewed each threat in terms of the pathway of effects from stressor to individual, starting with studies on beaked whales, but relying on inference from other cetacean species when no beaked whale-specific literature existed. We used an iterative snowball method to source literature for our review and identify evidence of impacts on beaked whales, using the bibliography from the IUCN [10] Red List assessment for each beaked whale species as a ‘start set’. Where available we reviewed the original citations, which included peer-reviewed publications, government research documents and other grey literature reports. We then reviewed the citations contained within each source for additional references. When no studies were identified for a species, we conducted individual Google Scholar keyword searches for the beaked whale species [common and Latin name] AND [threat], to ensure we included any potential papers that may have been missed. We documented all published studies found (both original sources and review papers) that mentioned or measured impacts to beaked whales published up to August 2023 (electronic supplementary material, table S1).

There are various methods for assessing and ranking human threats to wildlife, which can be based on species-specific traits, vulnerability scores, spatial overlap and/or the potential for population-level consequences [10,13,14]. For most beaked whale species there are large knowledge gaps about the extent and status of populations. Therefore, we did not assess threats at the population level, as effects will vary depending on population size and geographical context. Instead, we focused our assessment on evidence for the level of impact (i.e. severity) of each threat to individual animals. While integrating lethal or sub-lethal stressors in population-level assessments remains challenging, and depends on the nature of the threat, exposure and context, understanding direct effects on individuals is a critical component of assessing cumulative or population-level impacts [15–17].

Although threats were defined and assessed separately based on the pathway of effects [18], we acknowledge that threat categories will sometimes overlap. Alternative assessment frameworks or threat definitions may combine threats we have distinguished here (e.g. multiple stressors can originate from vessels; noise can also be defined as ‘pollution’).

Impact level was assessed based on a scale of severity of known effects, ranging from Serious (i.e. directly linked to mortality) to Moderate (i.e. behavioural disturbance and stress). The impact of each threat was informed by available evidence identified for beaked whales. In some cases, the mechanistic understanding of the pathways of effects was also supported by studies of other cetaceans.

We defined the level of impact for each threat as either:

— UNKNOWN—the threat is presumed to have an effect, but the level of severity for beaked whales is presently unknown.
— SERIOUS—the threat has been associated with the mortality of one or more individuals.
— INTERMEDIATE—the threat has been associated with injury or harm directly affecting physical health or reproduction in one or more individuals.
— MODERATE—the threat has been associated with disturbance or increased stress to one or more individuals.

## 3. Threats

### 3.1. Climate change

Global climate change is drastically altering marine ecosystems, with rising sea levels, increased ocean acidification, more frequent and intense marine heatwaves, decreased sea ice and impacts on oceanic carbon sequestration. These alterations have significant implications for the migration, growth, reproduction and survival of marine organisms, including cetaceans [19]. Albouy *et al*. [20] assessed the vulnerability of beaked whales to climate change, finding that several species show higher sensitivity and vulnerability compared with other marine mammals. Key traits contributing to the sensitivity of beaked whales include their diet specialization, restricted geographic ranges or ranges that span latitudinal gradients, long generation times, low reproductive rates and large body sizes [20].

Shifts in cetacean distribution associated with climate change are increasingly evident worldwide due to altered prey resources and rising ocean temperatures [21,22]. Predictive studies indicate that beaked whales, along with other cetaceans, are likely to experience continued range shifts towards higher latitudes, resulting in reduced suitable habitats [23–28]. These changes could exacerbate other stressors faced by beaked whales and lead to new challenges, such as increased fishing pressures, altered noise exposure due to new shipping routes and heightened disease outbreaks [29–31]. The complex pathways and cumulative often indirect nature of climate change effects have been demonstrated by recent mortalities of North Atlantic right whales (*Eubalaena glacialis*), who shifted their migration and foraging areas due to altered prey resources and increased their risk of vessel strike [32–34] and entanglement. The intersection of climate change with all other threats emphasizes the need to adopt flexible and adaptive conservation strategies for cetaceans [35,36].

***Level of impact***: UNKNOWN.

**Figure 1.**
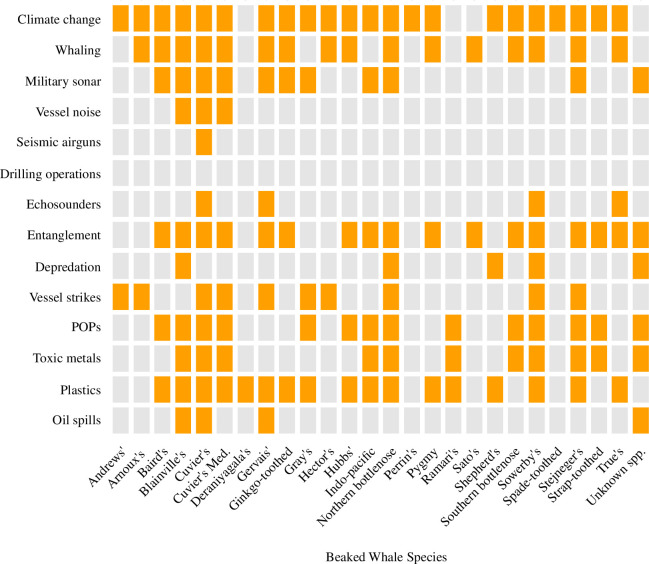
Summary of the impacts of 14 threats on beaked whale species based on available supporting publications identified in this review. Orange square indicates there are one or more studies. The Mediterranean population of Cuvier’s beaked whales (Cuvier’s Med.) was assessed separately in this review based on their IUCN status. Electronic supplementary material, table S1 provides the supporting references. POPs, persistent organic pollutants.

***Available information***: Available studies identify 22 species of beaked whales as highly likely to be affected by climate change, based on predictions from trait-based or environmental modelling (figure 1, electronic supplementary material, table S1).

***Rationale***: The specific pathways of effects of climate change on beaked whales are difficult to observe or predict due to a paucity of information on the ecology and life history of most species. Thus, the potential adverse effects on individuals remain largely unknown, and may be indirect, multifaceted and interconnected with other existing or emerging stressors. The effects of climate change on beaked whales are likely to become more apparent at the population level and when examined over longer time scales.

### 3.2. Whaling and directed take

Industrial whaling in the North Atlantic during the nineteenth and twentieth centuries led to significant declines in large whale populations, with profound yet poorly understood ecosystem effects [37–39]. Studies suggest that the widespread removal of large marine predators has affected ecosystems, including carbon and nutrient cycling, potentially triggering regime shifts and impacting resilience to environmental changes [40–43].

While northern bottlenose whales and Baird’s beaked whales were the primary targets of commercial whaling, at least 13 other beaked whale species have been hunted opportunistically or for ‘scientific research’ (figure 1, electronic supplementary material, table S1). Baird’s beaked whales continue to be hunted commercially, with unclear population impacts [9], whereas the commercial hunt for northern bottlenose whales, which harvested over 65 000 individuals, ceased in the 1970s [44,45]. A small-scale hunt for northern bottlenose whales in the Faroe Islands persists with minimal annual takes [46].

The legacy of historical whaling, particularly for northern bottlenose whales, along with the ongoing whaling of Baird’s beaked whales, presents ongoing challenges for the conservation of these species. Geographic isolation and culturally transmitted behaviours may lead to variable recovery rates across sub-populations, underlining the complexity of assessing and managing the long-term effects of whaling and directed take on beaked whale species [47].

***Level of impact**:* SERIOUS.

***Available information***: The effects of whaling have been well documented, and 15 species of beaked whales have been or are currently subjected to directed take (figure 1, electronic supplementary material, table S1).

***Rationale***: The killing of one or more individuals provides a clear pathway of direct effects on individuals, resulting in injury and death. Commercial whaling also reduced whale numbers worldwide, disrupting marine ecosystems and the social structure of populations on which individuals depend.

### 3.3. Acoustic disturbance

Beaked whales rely on sound for essential life functions including finding prey, communicating and sensing their environment. Human activities, including vessel traffic, oil and gas operations, military exercises, low-level aircraft, construction and marine acoustic technologies, contribute significantly to ocean noise [48,49]. Adverse effects of noise on cetaceans can be categorized as physiological (e.g. hearing impairment, stress, organ damage and mortality), behavioural (e.g. disruption of foraging and socializing, displacement and stranding) and ecological (e.g. indirect effects to prey and auditory masking) [50]. However, there are still major uncertainties regarding the extent of noise impacts, source characteristics, exposure levels and the potential for population-level consequences [8,51]. Here, we review current knowledge on five common sources of noise and their effects on beaked whales.

### 3.3.1. Military sonar

Military sonar use has been linked to fatal mass strandings of at least eight beaked whale species worldwide [52–54]. Stranded animals showed symptoms similar to decompression sickness, including gas bubble lesions and fat emboli in blood vessels and organs, probably due to altered diving behaviour and a physiological ‘fight or flight’ response to sonar exposure [55]. Controlled exposure experiments have shown that beaked whales exhibit strong avoidance behaviours when exposed to sonar, including extended dive durations, rapid movement away from the sound source and cessation of foraging (e.g. [56,57]). Cuvier’s and Blainville’s beaked whales have been the primary focus of experimental work, but similar responses have been observed in Baird’s beaked whales [58] and northern bottlenose whales [59,60]. These findings suggest that whales in areas with infrequent sonar use may perceive even distant sonar as a threat, highlighting the importance of exposure context.

Mass strandings associated with military exercises have occurred in various regions, such as the Canary Islands in 1988 [61], the Mediterranean Sea in 1996 [62,63], the North Pacific in 1994 [64], the Bahamas in 2000 [65] and the western Pacific between 2004 and 2019 [53,54]. Although not conclusively linked to military sonar, in 2018, multiple beaked whale species were stranded across the British Isles, and northern bottlenose whales were stranded in Iceland, coinciding with a NATO training exercise conducted in the Norwegian Sea [66,67]. It is important to note that beaked whale strandings are more likely to be detected near populated coastlines, while the effects of offshore naval exercises are difficult to observe and cryptic mortality is likely [68].

Sub-lethal effects are similarly challenging to observe, but experimental research has demonstrated that exposure to sonar can significantly disrupt normal behaviours in beaked whales, leading to altered dive cycles, the cessation of foraging activity over sustained periods, and long-range displacements from an affected area [56,59,69,70]. This scale of disturbance is likely to incur energetic costs and may result in a loss of foraging opportunities, underscoring the need for careful consideration of sonar use in marine environments [69,71].

***Level of impact**:* SERIOUS.

***Available information***: At least 11 species of beaked whales have been found to be highly sensitive to sonar either in experimental behavioural response studies and/or individual or mass strandings conclusively linked to military sonar use (figure 1, electronic supplementary material, table S1).

***Rationale***: The pathway of effects of exposure to military sonar on beaked whales clearly indicates strong behavioural responses that can result in the death of multiple individuals. Avoidance responses to sonar are probably context specific, but observed responses include the cessation of foraging, altered diving behaviour and displacement from large areas, which would probably result in energetic costs and lead to reduced health of individuals.

### 3.3.2. Vessel noise

Increasing marine traffic has led to a rise in low-frequency ambient ocean noise globally [72]. Little is known about the effects of vessel noise on most beaked whale species. While the auditory masking effects of vessel noise are of greatest concern for baleen whales, which produce calls within the same frequency range as the peak sound energy emitted by large vessels (10 Hz–1 kHz), fast-moving vessels can also generate significant sound energy at higher frequencies (greater than 10 kHz) [73]. Beaked whales produce echolocation clicks with peak frequencies ranging from 16 (Baird’s beaked whales) to 66 kHz (Sowerby’s beaked whales, BW70) [74,75] and may experience some degree of auditory masking by the high-frequency components of vessel noise, particularly at close ranges.

Studies have indicated that Cuvier’s beaked whales in the Mediterranean Sea may avoid high-traffic areas, and observations of both Cuvier’s and Blainville’s beaked whales have shown behavioural responses to vessel noise, leading to short-term impacts on foraging efficiency [6,76,77]. These responses appear to be less acute than reactions to sonar, but could cumulatively affect foraging energetics if behavioural disruption is chronic. While many beaked whale species avoid vessels, northern bottlenose whales have a propensity to approach vessels [78], though it is unclear if this behaviour, which could increase the probability of other harmful effects (e.g. vessel strike and entanglement), is triggered by vessel noise.

Vessel noise is pervasive in the marine environment, and chronic exposure to noise has been found to trigger physiological stress responses in humans (e.g. [79]), birds (e.g. [80]) and cetaceans [81], among many other taxa. Increased physiological stress occurring repeatedly or over prolonged time periods is known to affect fitness [82,83], and chronic stress in humans can lead to adverse health effects including increased risk of cardiovascular disease [84] and reduced immune function [85]. Studying the effects of chronic exposure to noise on the physiological health of wild cetaceans remains exceedingly challenging; however, anthropogenic noise is increasingly being incorporated into population models as an important stressor affecting cetacean populations [86,87].

***Level of impact**:* MODERATE.

***Available information***: There is limited evidence of direct behavioural responses to vessel noise in at least two species of beaked whales (figure 1, electronic supplementary material, table S1), and very limited information on the potential effects of chronic noise exposure on other cetaceans.

***Rationale***: The pathway of effects for vessel noise indicates it has the potential to cause behavioural responses and increased stress in beaked whales. Avoidance, disturbance and stress responses could result in a loss of foraging opportunities, reduced foraging efficiency and potentially decreased health in individuals.

### 3.3.3. Seismic air guns

Seismic air guns, extensively used in exploring sub-seafloor geophysical features like oil and gas reserves, are major contributors to marine noise pollution, emitting intense sound pulses at high source levels for extended periods of time. The dominant acoustic energy produced by seismic air guns is in the frequency range of 10–120 Hz, but broadband sound energy can also be produced up to frequencies of 22 kHz or higher [88,89]. Potential effects of seismic air guns on cetaceans include physiological or auditory injury, chronic stress and behavioural changes [90]. Limited experimental research exists on the effects that may be specific to beaked whales, but studies on other odontocetes, including harbour porpoises (*Phocoena phocoena*), sperm whales (*Physeter macrocephalus*), pilot whales (*Globicephala macrorynchus*) and Atlantic spotted dolphins (*Stenella frontalis*) have observed short-term behavioural responses ranging from varying degrees of displacement, avoidance or alteration of foraging behaviour [91–94]. Broad-scale multi-species studies have indicated significant decreases in odontocete sightings during seismic surveys [95,96]. Any disruption to normal diving behaviour is likely to have energetic consequences for beaked whales due to the energetic constraints of performing deep dives; these species may also experience higher received noise levels as they enter deep sound channels [89].

One documented stranding of two Cuvier’s beaked whales occurred in Mexico in 2002 during a nearby seismic air-gun survey [97]. Barlow and Gisiner [98] later noted that 3.5 kHz echo-sounders, similar in frequency to naval sonars, were used concurrently with the seismic air guns during this survey, and the specific cause of the beaked whale stranding remains unknown. The probability of observing harmful effects from seismic surveys, including mortality, is low in offshore areas of beaked whale habitat.

***Level of impact**:* UNKNOWN.

***Available information***: There are limited available data on the potential effects of noise generated by seismic air-gun surveys on cetaceans. There is one documented observation of a stranding of Cuvier’s beaked whales coincident with seismic surveys, but no conclusive link was made (figure 1, electronic supplementary material, table S1).

***Rationale***: The pathway of effects for exposure to seismic air guns suggests some cetacean species exhibit a behavioural response, including avoidance and short-term displacement, which may be context specific. Such responses in beaked whales could potentially lead to a loss of foraging opportunities, reduced health or more substantial consequences. Current evidence linking seismic air guns to adverse effects for beaked whales is anecdotal and further research is needed.

### 3.3.4. Drilling operations

In addition to geophysical surveys using seismic air guns, a variety of other noise-producing activities are associated with offshore oil and gas development. Offshore drilling operations conducted from fixed platforms generally produce appreciable levels of low- to mid-frequency noise [99,100]. Drilling from drill ships or mobile units produces higher levels of noise, due to the dynamic positioning thrusters used to maintain the ship’s position throughout the operation [100]. Sound source characterization studies conducted during two different exploratory drilling projects occurring off the Scotian Shelf in eastern Canada found that the highest noise levels were produced by the dynamic positioning thrusters [101,102]. Other sources of noise during these operations included noise from the drill bit and string, noise from generators and other machinery on board the drill ship and support vessels, and higher-frequency pings from locator beacons [102]. There is no specific information available on the effects of noise associated with drilling operations on beaked whales, and data are very limited for other species of cetaceans [103], but the effects may be similar to those caused by vessel noise and seismic air-gun surveys. Exposure will generally be more localized for drilling operations, which are stationary.

***Level of impact**:* UNKNOWN.

***Available information***: There is no information available to assess the effects of noise from drilling operations on beaked whales, and inconclusive data on the effects of this source of noise on other cetaceans (figure 1).

***Rationale***: The pathway of effects for the impacts of drilling activities on beaked whales is unclear. These activities are known to generate non-trivial sources of noise and contribute to the cumulative anthropogenic noise present in the oceans. More research is needed to understand whether and how noise from drilling operations may adversely affect beaked whale species.

### 3.3.5. Echo-sounders

Active acoustic technologies, including depth sounders, acoustic sub-bottom profiling systems, commercial fish-finders and scientific echo-sounders, contribute to the marine soundscape, potentially affecting beaked whale species. While this broad range of technologies typically operate at higher frequencies and ensonify smaller areas than military sonars, they still have an effect. Multi-beam echo-sounders, especially those used in hydrographic seafloor mapping, emit sound over a wide swathe, sometimes using low frequencies (e.g. 12 kHz) in deep waters. While there is a potentially lower probability of direct auditory injury compared with military sonars or seismic air guns, the effect of echo-sounders on cetaceans and the potential for adverse behavioural responses remains unclear [104].

Studies have shown varying responses of beaked whales to these technologies. Cholewiak *et al*. [105] observed a significant reduction in beaked whale click detections on a towed hydrophone array during active pinging of shipboard scientific echo-sounders, indicating potential behavioural changes like avoidance or altered foraging. Conversely, a study by Kates Varghese *et al*. [106] on Cuvier’s beaked whales found no consistent behavioural response during a seafloor mapping survey using a 12 kHz multi-beam echo-sounder, though this occurred on the Southern California Antisubmarine Warfare Range, an area of frequent sonar use. Other odontocetes, such as short-finned pilot whales, have shown reactions to scientific echo-sounders [107]. A notable incident involving melon-headed whales (*Peponocephala electra*) stranding in Madagascar was linked to the use of a high-powered 12 kHz multi-beam echo-sounder [108]. These findings highlight the complex nature of cetacean responses to acoustic technologies, which are widely used and typically occur in conjunction with other sources of anthropogenic noise, such as vessels and seismic air-gun surveys.

***Level of impact***: MODERATE.

***Available information***: There is evidence of behavioural responses to echo-sounders in at least four species of beaked whales, as well as other odontocetes, including avoidance and altered foraging behaviour (figure 1, electronic supplementary material, table S1).

***Rationale***: The pathway of effects for echo-sounders indicates exposure has the potential for a behavioural response in some beaked whale species and contexts. Avoidance and disturbance responses could result in a loss of foraging opportunities, reduced foraging efficiency and potentially decreased health in affected individuals.

### 3.4. Fisheries interactions

Fishery impacts, including injury or death due to entanglement in fishing gear, are widely regarded as one of the most severe threats to cetaceans [109]. Direct effects may occur incidentally when whales are foraging in the same area or on the same species targeted by fisheries, or due to learned associations with fisheries for food (i.e. depredation behaviour). It has been demonstrated that where cetaceans and fisheries coincide, outcomes can range from prey supplementation increasing whale populations [110] to fishers killing marine mammals to deter depredation and reduce competition with fisheries [111]. Indirect ecosystem impacts of fisheries may also deplete prey availability for cetaceans, although the potential broad-scale indirect effects of fisheries are poorly understood and were not assessed here. However, as the ongoing global expansion of fisheries continues, the potential of direct and indirect effects will probably increase [112].

### 3.4.1. Entanglement

Entanglement, defined here as the incidental capture of cetaceans in fishing gear (e.g. ropes, lines, nets or hooks), can often lead to death, injury or compromised health and reproduction [113,114]. Despite widespread recognition of entanglement as a major conservation concern for cetaceans, its impact on beaked whale species is poorly understood [109]. Entanglements have been documented globally for at least 15 beaked whale species (figure 1, electronic supplementary material, table S1); however, many incidents probably go unreported, due to low rates of self-reporting by fishers, few at-sea fisheries observers in offshore areas, and potential misidentification (e.g. [115], but see [116]). Entanglement of beaked whales appears to be relatively less common than coastal cetaceans [117]; however given the challenges, differences in reporting may be biased by effort. In addition, larger species (e.g. *Hyperoodon*, *Ziphius* and *Berardius* spp.) may be strong enough to break free and swim away with or without gear before an entanglement is recorded. Entanglement can also lead fishers to retaliate against cetaceans, engage in negative deterrents or incentivize directed take [109].

Evidence of entanglement can also come from observations of scars and injuries on whales, indicative of previous encounters with fishing gear [118,119]. Feyrer *et al*. [119] found a consistent rate of anthropogenic scarring on northern bottlenose whales, with males more frequently affected. However, interpreting these scars on other beaked whale species may be challenging due to the potential overlap with conspecific scarring [120]. While beaked whale strandings offer valuable data, the offshore habitat of beaked whales means that carcasses seldom reach coastal areas for examination, limiting our understanding of fisheries-related mortality [121–123]. Consequently, existing records from strandings, observer reports and scarring probably represent only a fraction of actual entanglement incidents.

***Level of impact**:* SERIOUS.

***Available information***: Entanglements have been attributed to multiple mortalities and injuries for at least 15 species of beaked whales (figure 1, electronic supplementary material, table S1). Injuries have been linked to long-term health effects in other species of cetaceans. Gear loss, lack of observer coverage or negative incentives for entanglement reporting probably affect available information.

***Rationale***: The pathway of effects for entanglement can lead to death by drowning, lethal and sub-lethal injuries from gear and for individuals that survive, reduced health and reproduction.

### 3.4.2. Depredation

Depredation occurs when whales learn to remove fish from fishing gear or feed on escapees and discards from fisheries [109,124]. Cetaceans engaging in depredation behaviour may face an increased risk of injury or mortality due to close associations with vessels and gear, or deterrent methods that can be employed by fishers [110]. Depredation behaviour has been documented in at least 19 species of odontocetes across the globe, and commonly occurs with long-line fishing activities (e.g. 110,124,125); however, depredation on trawl and purse seine fisheries has also been observed [116,126,127]. Despite the potential for negative consequences, reduced foraging costs associated with depredation led to survival benefits and growth in some cetacean populations [125,128].

Beaked whale depredation behaviour has primarily been documented for northern bottlenose whales in trawl, gillnet and long-line fisheries [116,129–131], and there is some evidence of long-line depredation interactions (e.g. hooked in the mouth or stomach) occurring with Sowerby’s beaked whales [132], Shepherd’s beaked whales (*Tasmacetus shepherdi*) [133], Blainville’s beaked whales [134] and unidentified mesoplodonts [134]. Northern bottlenose whales have also been observed approaching fishing vessels and being hand-fed (intentional provisioning) by fishers [49]. For northern bottlenose whales, where depredation is established in some areas, the behaviour is likely to increase due to the positive energetic benefits and social nature of this species [135].

***Level of impact*** **:** SERIOUS.

***Available information*** **:** Direct and indirect evidence indicates at least five species of beaked whales engage in at least occasional depredation behaviour (figure 1, electronic supplementary material, table S1).

***Rationale*** **:** The pathway of effects for depredation starts with a learned behavioural response to the presence of fisheries. This response alters an individual’s natural foraging behaviour, increasing the potential for injuries and mortality from entanglements, ingestion of hooks or other gear, vessel strikes, retaliation by fishers and dependence on foraging in association with fisheries.

### 3.5. Vessel strikes

Vessel strikes can result in trauma-related injuries from the collision that may directly or indirectly lead to death. For whales that survive, injuries may have long-term adverse effects on health, reproduction and ultimately fitness, and can result in population-level consequences [136]. As the number of commercial and recreational vessels increase throughout the world’s oceans, so does the risk of vessel strike. While reports of vessel strikes are more commonly associated with large baleen whale species, vessel strikes involving beaked whales are known to occur. Schoeman *et al.*’s [136] global review of marine animal vessel collisions identified eight different beaked whale species involved in vessel strike incidents. In addition, Feyrer *et al.* [119] documented vessel propeller trauma on live northern bottlenose whales from scar patterns (e.g. fin amputation and large gashes in the spine), which were not included in Schoeman *et al.*’s [136] review. Northern bottlenose whales are known for their curious nature and often approach and follow vessels [44]. Propeller vessel strike injuries are common in species that approach vessels, bow-ride [137] or swim in the wash of the propellers [138].

Lower vessel density and fishery observer coverage in offshore areas of beaked whale habitat decreases the probability that incidents, injuries or carcasses will be seen and reported [139]. In addition, injuries that are not externally apparent (such as bruising and fractures) limit our ability to estimate the full impact of vessel strikes.

***Level of impact***: SERIOUS.

***Available information*** **:** Vessel strikes have affected at least nine species of beaked whales, with evidence of mortalities, severe injuries and scars (figure 1, electronic supplementary material, table S1).

***Rationale*** **:** The pathway of effects for vessel strikes can result in blunt force trauma leading to death, lethal and sub-lethal injuries and for individuals that survive, reduced health and reproduction.

### 3.6. Pollution and chemical contaminants

There are a range of harmful direct and indirect effects associated with chemical and physical pollutants in the marine environment. Potential effects depend on factors like concentration, prolonged exposure, degradation and interaction with other pollutants, and long-term effects are often highly uncertain [140–142]. This review focuses on threats from contaminants and pollution sources known to harm marine life, including persistent organic pollutants (POPs), metals, oil and plastic debris.

Pollutants can enter the marine environment and animal tissues through various contemporary, historical or unknown sources, which may only be traced after a significant exposure level or health effect has been identified. Direct sources may include litter, wastewater, shipping activities, military dumpsites, oil and gas development, and fishing debris. Indirect sources can involve atmospheric industrial emissions and particulate matter, as well as degradation or weathering of direct deposits. Due to the diversity of contaminants, studies are typically highly specific and will focus on a group of chemical pollutants (e.g. polychlorinated biphenyls (PCBs), pesticides or plastics) or similar sources (e.g. spills). Given limited data for beaked whales, this assessment characterizes the threats posed by the most well-studied pollutants: POPs, including pesticides (e.g. dichlorodiphenyltrichloroethane (DDT)), metals, oil spills and plastics.

### 3.6.1. Persistent organic pollutants

POPs are toxic, long-lasting chemicals that accumulate in the fatty tissues of animals and biomagnify within food chains [143–145]. While most POPs were banned by the 1980s, leading to the Stockholm Convention in 2001 [146], marine mammals remain vulnerable due to their long lifespans, top predator status, large blubber reserves and reduced metabolic capacity to break down these chemicals [147,148]. POPs include PCBs, organochlorine pesticides, such as DDT, chlordane, dieldrin, toxaphene, hexachlorobenzene, hexachlorocyclohexane and polybrominated diphenyl ethers.

Marine mammals inhabiting contaminated waters have suffered adverse effects on reproduction, immunity, carcinogenicity and ultimately survival and population growth [149–154]. Contaminant concentrations may vary by sex, age, species, migratory behaviour and diet, reflecting diverse pathways and vulnerabilities to exposure. Beaked whales, feeding on deep-water prey, may ingest intermediate to high contaminant loads as mesopelagic fish and deep-sea squid are thought to be sinks for POPs [155,156].

Due to the difficulties of conducting controlled exposure studies of cetaceans, toxicity thresholds for most POPs have not been established. However, experimental studies of seals, and wild populations of cetaceans living in contaminated areas have highlighted the adverse health effects of PCBs [149,151,157,158]. PCB toxicity thresholds range from 17 µg g^−1^ lipid weight for immune and reproductive effects to 41 µg g^−1^ for reproductive impairment, with physiological cellular changes observed at concentrations as low as 1.3 µg g^−1^ lipid weight, indicating a lower threshold for health effects [159–161].

Research on PCBs and DDT in beaked whales, while derived mostly from older, demographically limited studies, indicate PCB levels can exceed thresholds [162,163], with average concentrations around 7.16 μg g^−1^ (lipid weight ± s.d., figure 1, electronic supplementary material, tables S1 and S2). Stejneger’s beaked whales (*Mesoplodon stejnegeri*) in the Sea of Japan exhibited PCB levels exceeding the lower health effect threshold (17 µg g^−1^), and some male Cuvier’s beaked whales in the Mediterranean had PCB levels above the higher threshold for reproductive impairment (41 µg g^−1^) [162,163]. Additionally, northern bottlenose whales from the Scotian Shelf exhibited higher POP levels, including DDT, than their Arctic counterparts [164,165].

***Level of impact**:* INTERMEDIATE.

***Available information***: At least 12 species of beaked whales have been found with some concentration of POPs in their tissues (figure 1, electronic supplementary material, tables S1 and S2). For a few individuals, PCB levels were found to surpass thresholds for health effects and reproductive impairment. The average concentration of PCBs across beaked whale specimens reviewed was above the biological molecular toxicity threshold suggesting that PCBs may be affecting physiology at a molecular and cellular level. However, data on thresholds and effects of POP concentrations for cetaceans or beaked whale species is limited.

***Rationale***: The pathway of effects for exposure to POPs can result in reduced health and reproductive success of individuals.

### 3.6.2. Metals

Heavy metals like mercury, cadmium, lead and arsenic, present at low natural levels in the marine environment, can become toxic due to elevated concentrations from anthropogenic sources, such as industrial emissions, mining and agricultural run-off [141,166,167]. Globally, heavy metal concentrations have been increasing since pre-industrial times [168]. While heavy metals are primarily found in sediments, they can become entrained in marine waters due to disturbance or as part of oceanographic circulation [166,169].

Ingestion of metals can potentially affect marine life even at low levels, though our understanding of toxicity thresholds for metals in cetaceans is limited [170]. Complexities arise from chemical interactions (occurring between metals and other contaminants) and varying tissue-specific concentration patterns [171]. Marine mammals have adaptations to metabolize or detoxify higher concentrations of certain metals, complicating the interpretation of health effects based on concentrations toxic to terrestrial mammals or humans (e.g. selenium, mercury [172,173]). Despite the uncertainties, the toxicity and health effects of some metals, particularly, mercury and cadmium, have been well studied for at-risk cetacean populations. For example, high mercury concentrations in beluga whales (*Delphinapterus leucas*) have been linked to cellular and neurological damage, kidney dysfunction and immune system impairment [172].

Studies describing metal concentrations in tissues exist for at least nine beaked whale species, providing some data on their exposure to potentially toxic metals. However, these studies are limited in scope (i.e. small sample sizes, restricted to stranded individuals and large variability across tissue types), making broader spatial or temporal generalizations and identification of health effects difficult (figure 1, electronic supplementary material, tables S1 and S2).

***Level of impact:*** INTERMEDIATE.

***Available information***: There are at least nine species of beaked whales where metals were found in detectable concentrations in various tissues (figure 1, electronic supplementary material, tables S1 and S2). While there is a range of uncertainty on their effects in cetaceans, for some well-studied metals (e.g. mercury), toxicity has been established. Many metals are linked to adverse health effects at any level or concentration. There are no studies of the effects of metal toxicity on beaked whales.

***Rationale***: The pathway of effects for metal toxicity in beaked whales can potentially result in the reduced health and reproductive success of individuals.

### 3.6.3. Plastics

Reported instances of cetaceans ingesting plastic debris have risen steadily since 1960, including in rarely observed beaked whales [174]. Consuming plastic can result in digestive blockages, nutritional issues, infections and starvation [175]. Plastic bags or sheets are commonly observed in beaked whale stomach contents, and a variety of other items including fishing gear, bottles, packaging, cigarette butts and microbeads have been found (electronic supplementary material, table S2); all of which will eventually degrade into microplastics (less than 5 mm) and nanoplastics (1–1000 nm) [176–178]. Although marine debris may drift offshore by air or sea from coastal sources (e.g. helium balloons and plastic bags), fishing activities are considered the primary source in remote regions inhabited by beaked whales [179–181].

We found at least 16 species of beaked whales with reports of plastics in their digestive tracts (figure 1, electronic supplementary material, table S1). Modern whalers first reported plastic bags and other items in the stomachs of northern bottlenose whales in 1967 [182]. It has been suggested that deep-diving species potentially ingest more plastic than pelagic species, because they may mistake plastic bags for cephalopods [180,183]. Although 30% of incidents in our review merely mention finding unspecified plastic, of records where plastic items were identified, nearly 50% were specified as bags or ‘sheets’ (electronic supplementary material, table S2 ). We also noted where plastic ingestion by beaked whales was documented in association with digestive health conditions like gastritis, haemorrhages, ulcers, perforation and parasitic infections [184–191].

Microplastics are probably consumed by cetaceans either directly or by their prey, and have been found in northern bottlenose, True’s (*Mesoplodon mirus*) and Cuvier’s beaked whales [180,181]. As a source of exposure for other contaminants (e.g. POPs), microplastics may contribute to bioaccumulation and exacerbate adverse health effects [177,180,192].

Reports of plastic consumption by beaked whales primarily come from stranded animals, which could have a higher likelihood of having ingested plastics, skewing data towards an overrepresentation of plastic impacts [180]. Conversely, stomach content analyses can overlook smaller particles and non-biological items, like plastic. Further research is needed to determine the prevalence and long-term effects of macro- and micro-plastic ingestion on beaked whales.

***Level of impact***: SERIOUS.

***Available information***: At least 16 species of beaked whales have been found to be affected by plastic ingestion on postmortem investigation, and plastic ingestion has been linked to starvation and death in multiple documented cases (figure 1, electronic supplementary material, tables S1 and S2).

***Rationale***: The pathway of effects for plastic pollution is primarily through direct ingestion. Ingesting plastics can result in a range of negative health effects (e.g. nutritional deficiencies, inflammation and blockages), which can lead to behavioural change and death. Direct or indirect microplastic ingestion can also increase exposure to other contaminants (e.g. POPs) and potentially lead to additional adverse effects.

### 3.6.4. Oil spills

Oil and gas activities, including development, extraction and transportation of petroleum products in marine ecosystems, pose significant risks of spills from vessels, offshore platforms, drilling rigs, wells or pipelines. Oil spills, which may be caused by collisions, groundings, fires, hull or equipment failures, heavy weather and human error, can have severe impacts on marine life. While large spills (>greater than 7 tonnes) are rare but of primary concern, the majority of spills are small and often go unreported, contributing to overall marine pollution [193]. Our review focused on the effects of crude oil spills, which are the most well documented. However, other oil products and chemicals related to extraction and clean-up are also a concern.

There is inadequate evidence that cetaceans are known to avoid crude oil, and whales have been observed swimming through surface oil after the Exxon Valdez spill (1989) and the Deepwater Horizon (DWH) spill (2010) [194,195]. As petroleum products are known to be toxic even at low levels, if whales are present during a spill it should be assumed that they will be directly affected by ingesting or inhaling oil or dispersants [196].

The impact of oil spills on cetaceans largely depends on the type of oil, volume, location and sea conditions [197]. However, the toxicity of direct exposure to oil slicks can result in fatal or long-term health issues, including lung disease, reproductive failure, stress and immune dysfunction. Because cetaceans breathe at the surface, where oil and volatile organic compounds (VOCs) are most concentrated and become aerosolized, their skin, eyes and respiratory systems are common pathways for exposure. The unique nature of cetacean respiratory physiology means that toxic VOCs and oil are not filtered during inhalation and are absorbed directly into the blood through the lungs [198]. The use of deep dispersants, which causes oil to sink, can also increase impacts on the benthos, which would potentially affect beaked whales as they forage at depth [196]. Ingestion of contaminated prey is another potential exposure pathway [197]. Social species with high site fidelity are more likely to be affected by spills in important habitat areas, which can also leave a legacy of lasting contamination on the local ecosystem [198,199].

The DWH spill is thought to have affected at least four species of beaked whales, contaminating their habitat, water, air and prey [200]. Studies during and after the spill revealed the presence and likely exposure of species including Cuvier’s, Gervais (*Mesoplodon europaeus*) and an unknown species of beaked whale (BWG) to oil and dispersants [201]. The patchy distribution and low overall density of beaked whales mean that the geographic extent of a spill can differ from other cetaceans and may not align with modelled predictions based on a uniform distribution (e.g. [139,202]).

***Level of impact**:* SERIOUS.

***Available information***: At least four species of beaked whales are known to have been affected by large oil spills in their habitat, despite a low probability of detection (figure 1, electronic supplementary material, table S1). Oil and its components are well known to be toxic to a wide range of marine life, and the adverse effects of exposure to large oil spills are probably the same for all cetaceans, including beaked whales. However, there are few studies on the effects of more frequent small spills.

***Assessment rationale***: The pathway of effects for direct and indirect exposure to large oil spills occurs through ingesting or inhaling petrochemicals, which, due to the acute toxicity of these substances, can lead to mortalities, and short- to long-term reductions in health and reproduction. The effects of small spills will vary depending on the exposure context (e.g. location, size, frequency, environmental conditions and composition of oil products), but if an individual is exposed, effects could be similar to larger spills.

## 4. Other potential threats

There are other potential stressors that were not included in our review due to limited available information on the pathway of effects to beaked whales or other cetaceans. Potential areas for future research include the relationship between stressors and reduced biological resilience, such as small population sizes, inbreeding and zoonotic diseases. Additionally, there are many human activities in the marine environment for which impacts and adverse effects are anticipated, but not fully understood. These include deep-sea mining [203], expanding mesopelagic fisheries [204], marine energy generation [205], offshore industrial activities involving pile driving [206], legacy of toxic dump sites [207], unregulated acoustic technologies such as ultrasonic antifouling devices [208] and potentially others of which we are not yet aware. In addition, impacts on beaked whale habitat and indirect effects, such as human-induced ecosystem changes affecting beaked whale prey, are difficult to study but should be considered as part of the broader context when interpreting this threat assessment.

## 5. Cumulative effects

Interactions between multiple stressors are complex and the effects are not well understood for most cetaceans, though our understanding is slowly improving [15–17,209]. The cumulative impacts faced by individuals and populations may be localized or general, vary over space and time, and past threats may continue to influence present status (e.g. whaling [210]). Due to synergistic or antagonistic interactions, tipping points in nonlinear responses, or secondary effects due to impacts on other trophic levels, exposure to multiple stressors is unlikely to result in a simple additive combination of effects [15,16,209]. Although we present the total number of threats identified for each species, cumulative effects were not addressed in this review. However, our assessment highlights that, despite their remote habitat, beaked whales are being exposed to multiple anthropogenic stressors, potentially increasing their overall vulnerability. Additional research and theoretical frameworks (e.g. [15,16]) are needed to better understand, manage and mitigate cumulative effects across the marine environment.

## 6. Future work and research needs

As our knowledge increases, the effects of historical, contemporary and future human activities will need to be reassessed for potential impacts on beaked whales. However, to inform risk assessments, management measures and mitigation activities, beaked whale species would benefit from additional studies to address the following knowledge gaps:

— estimating the size of beaked whale populations which are currently unknown;
— understanding the relationship between individual-level effects and population-level consequences of stressors;
— assessing the spatiotemporal overlap between the occurrence of stressors and beaked whales, including cumulative effects; and
— understanding the current and future impacts of climate change and effects of other stressors within the context of climate change.

## 7. Summary and conclusions

This threat assessment covers a wide range of stressors to beaked whale species, while recognizing that other threats may emerge in the future. Of the threats reviewed, most have been reported to affect one or more beaked whale species (figure 1). All threats appear to have historical, current and potentially future impacts on beaked whale conservation. In this article, we could not find specific studies that assessed the effects of drilling noise on any beaked whale species, despite the occurrence of offshore oil and gas development in beaked whale habitats, and the impacts of this threat remain unknown. Considering the diversity of beaked whale species and the challenges in studying them, it is remarkable that every species has been identified as potentially affected by at least one type of threat. Based on the literature, it appears that Cuvier’s beaked whales face the largest number of threats (*n* = 12); however, this is probably an artefact of being the most widely studied species of beaked whale and having a cosmopolitan distribution [8]. The threats affecting the largest number of beaked whale species (based on the currently available information) include climate change (*n* = 21), plastic pollution (*n* = 16) and entanglement (*n* = 15).

Similar threat assessments have been conducted for individual species of beaked whales [10,211]; here, we take a multi-species approach to extend the scope and compare available evidence using simplified assessment criteria. Overall, whaling, military sonar, entanglement, depredation, vessel strike, plastic pollution and oil spills are all threats presenting a serious level of impact on beaked whales, while the effects of climate change, seismic air guns and drilling operations are unknown and require further study (table 1). Although we take a data-driven approach to our assessment, there is almost no ability to observe and record the effects of many stressors due to the remote offshore habitat of beaked whale species, and it is highly likely that mortalities, injuries and other adverse effects of human activities are under-reported. Given the inherent potential of certain threats to rapidly affect the stability of small, vulnerable or endangered populations, it may seem practical to focus on stressors having serious impacts on individuals. However, beaked whales are long-lived species and a stressor assessed as having an intermediate or moderate impact on individuals could have serious long-term effects at a population level. It was outside the scope of this review to assess population-level effects; however, this review can help inform process-driven approaches for assessing population-level consequences and understanding the impacts of multiple stressors on data-poor species and populations [15,16].

**Table 1.** Impact severity of 14 threats to beaked whales, assessed at the individual level.

Level of impact	threat
UNKNOWN	Climate change
	Seismic air guns
	Drilling operations
SERIOUS	Whaling and directed take
	Military sonar
	Entanglement
	Depredation
	Vessel strikes
	Plastics
	Oil spills
INTERMEDIATE	Persistent organic pollutants
	Metals
MODERATE	Vessel noise
	Echo-sounders

*Notes:* Impacts may vary at the population level.

Finally, while it is beyond the scope of this threat assessment to prioritize specific management actions, it is important to emphasize that there is inherent uncertainty in scientific assessments of impacts for data-poor species, such as beaked whales. Threat assessments are increasingly required as part of evidence-based science advice necessary to support the triage of management priorities. However, from a scientific perspective, a level of impact classified as ‘Moderate’ or ‘Unknown’ does not imply that the threat is negligible—we expect all threats assessed to have some adverse effects. All impact assessments typically identify the need for more data, but this should not preclude management actions aimed at mitigating or reducing the potential effect of a threat, even in the absence of further study. Doing nothing could still result in serious impacts, and retrospective future actions may be insufficient to recover long-lived cetacean species.

## Data Availability

All data are included in the electronic supplementary material [212].
